# Comparing Pupil Light Response Modulation between Saccade Planning and Working Memory

**DOI:** 10.5334/joc.33

**Published:** 2018-06-26

**Authors:** Chin-An Wang, Jeff Huang, Rachel Yep, Douglas P. Munoz

**Affiliations:** 1 Centre for Neuroscience Studies, Queen’s University, Kingston, Ontario, CA

**Keywords:** attention, saccade preparation, pupil constriction, pupil dilation, arousal

## Abstract

The signature of spatial attention effects has been demonstrated through saccade planning and working memory. Although saccade planning and working memory have been commonly linked to attention, the comparison of effects resulting from saccade planning and working memory is less explored. It has recently been shown that spatial attention interacts with local luminance at the attended location. When bright and dark patch stimuli are presented simultaneously in the periphery, thereby producing no change in global luminance, pupil size is nonetheless smaller when the locus of attention overlaps with the bright, compared to the dark patch stimulus (referred to as the local luminance modulation). Here, we used the local luminance modulation to directly compare the effects of saccade planning and spatial working memory. Participants were required to make a saccade towards a visual target location (visual-delay) or a remembered target location (memory-delay) after a variable delay, and the bright and dark patch stimuli were presented during the delay period between target onset and go signal. Greater pupil constriction was observed when the bright patch, compared to the dark patch, was spatially aligned with the target location in both tasks. However, the effects were diminished when there was no contingency implemented between the patch and target locations, particularly in the memory-delay task. Together, our results suggest the involvement of similar, but not identical, attentional mechanisms through saccade planning and working memory, and highlight a promising potential of local pupil luminance responses for probing visuospatial processing.

## Introduction

Spatial attention enables observers to select a particular location for preferential visual processing, resulting in faster and better processing of information at attended locations than at other locations (Carrasco, 2011; Maunsell, 2015; Moore and Zirnsak, 2017). These facilitatory effects are not exclusive to attention. Instead, the efficient extraction of information at the location prepared for saccades or remembered in working memory has also been observed (review: e.g., Awh and Jonides, 2001; Awh et al., 2006; Bisley, 2011; Kowler, 2011). Moreover, similar neural networks have been recruited during these cognitive operations (Ikkai and Curtis, 2011; Jerde et al., 2012), together suggesting the involvement of overlapping mechanisms among attention, saccade planning, and spatial working memory.

Pupil size, controlled by balanced activity between the parasympathetic and sympathetic systems, is greatly used to index various cognitive and neural processes (e.g., Beatty, 1982; Nassar et al., 2012; Eldar et al., 2013; de Gee et al., 2014; Ebitz and Platt, 2015; Joshi et al., 2016; Urai et al., 2017). The major function of pupil size change is to modulate the amount of light projected onto the retina, with a decrease in pupil size for luminance increase and increase in pupil size for luminance decrease (referred to as the *global* luminance modulation), to regulate the trade-off between image acuity and sensitivity (Woodhouse, 1975; Laughlin, 1992). Recently, it has been shown that spatial attention interacts with the luminance level at the selected location (Binda and Murray, 2015a Mathôt and Van der Stigchel, 2015). Participants, when confronted with bright and dark patch stimuli presented in the periphery, produce a smaller pupil size when their locus of attention overlaps with the bright stimulus as compared to the dark stimulus, even though the level of global luminance remains unchanged (Binda et al., 2013; see also in Mathôt et al., 2013; Binda et al., 2014; Mathôt et al., 2014; Binda and Murray, 2015b). Furthermore, the preparation of an eye movement towards a stimulus embedded in the bright background induces smaller pupil size than preparation of an eye movement towards a stimulus in the dark background (Mathôt et al., 2015). These results together suggest that pupil size is modulated by the luminance level at the location selected by spatial attention (referred to as the *local* luminance modulation) to potentially optimize the processing of information at the selected location.

**Figure 1:**
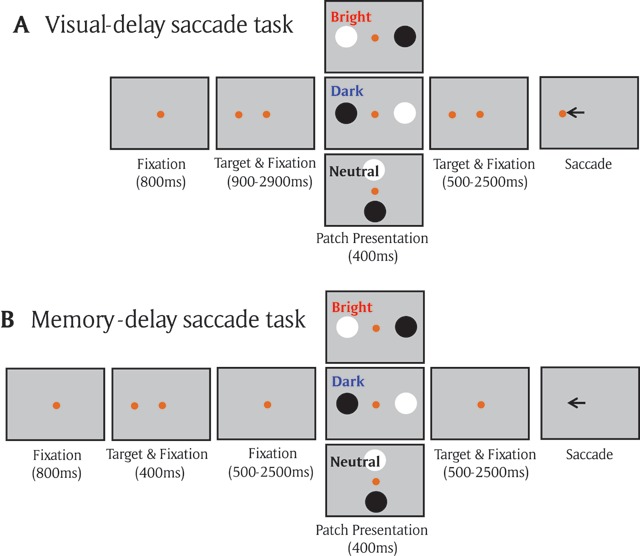
**A)** Each trial started with a central fixation point on a gray background. After a delay, there was a presentation of a target stimulus, and after a random delay the central fixation point disappeared and participants were required to move their eyes to the target. During the delay period, the two patch stimuli were presented briefly (400 ms), with the bright/dark patch being spatially aligned with the target location in the bright/dark condition, respectively. Neither patches were aligned with the target location in the neutral condition. **B)** Memory-delay task was similar to the visual-delay task except the target stimulus was only presented briefly (400 ms).

Although the interdependent relationship between attention and saccade planning or working memory has been developed separately, a direct comparison between the effects resulting from saccade planning and working memory remains less explored. If the consequence of saccade planning and working memory is comparable to that of a shift of spatial attention, then similar modulation between saccade programing and spatial working memory should be observed. Here, we use the local luminance modulation to directly examine the effects of saccade planning and spatial working memory. Participants were required to generate a saccade toward a visual target location (visual-delay saccade task) or a remembered target location (memory-delay saccade task). During the delay period, bright and dark patch stimuli were presented simultaneously, with either the bright or dark patch being spatially aligned with the location that was being prepared for an upcoming saccade destination or the location that was being remembered in working memory (Figure 1). We hypothesized that similar local luminance modulation should be observed between the two tasks, that is, larger pupil constriction after patch presentation when the bright patch, as compared with the dark patch, is spatially aligned with the target location.

## Materials and Methods

*Experimental setup*. All experimental procedures were reviewed and approved by the Ethics Board of Queen’s University and were in accordance with the principles of the Canadian Tri-Council Policy Statement (TCPS-2 2014) on Ethical Conduct for Research Involving Humans, and the Declaration of Helsinki (World Medical Association, 2001). Twenty participants in Experiment 1 (6 + 4 participants in Experiment 2) were recruited for this study (age range = 18 and 35). Participants had normal or corrected to normal vision and were naïve regarding the purpose of the experiment. They provided informed consent and were compensated for their participation. Eye position and pupil size were measured with a video-based eye tracker (Eyelink-1000 binocular-arm, SR Research, Osgoode, ON, Canada) at a rate of 500 Hz with binocular recording (left pupil was mainly used). Stimulus presentation and data acquisition were controlled by Eyelink Experiment Builder and Eyelink software. Stimuli were presented on a 17-inch LCD monitor at a screen resolution of 1280 × 1024 pixels (60 Hz refresh rate), subtending a viewing angle of 32° × 26°, with the distance from the eyes to the monitor set at 58 cm.

*Visual-delay saccade task (Exp 1: Figure 1A)*. Participants were seated in a dark room, and the experiment consisted of 165 trials. Each trial began with the appearance of a central fixation point (FP) (0.6° diameter; 10 cd/m^2^, isoluminant color to the background) on a gray background (10 cd/m^2^). After a period of 800 ms, a peripheral target (1° diameter; isoluminant to the background) appeared in one of 16 possible locations (8 different radial angles at 0, 45, 90, 135, 180, 225, 270, and 315°, each at two eccentricities of 4 or 8° visual angle from the central fixation spot). After a variable delay (900–2900 ms), two circular patches (4.5° in diameter, one bright and the other dark, both with 95% contrast relative to the gray background, referred to as the patch stimuli) were displayed for 400 ms. After another variable delay (500–2500 ms), the FP was removed, and participants were required to generate a saccade toward the target. Three types of patch stimulus conditions were used: in the bright condition (20% of trials), the bright patch stimulus location was spatially aligned with the target location and the dark patch was presented at the opposite location of the bright patch. In the dark condition (20% of trials), the dark patch stimulus location was spatially aligned with the target location and the bright patch was presented at the opposite location of the dark patch. In the neutral condition (60% of trials), neither of the patch stimuli locations were spatially aligned with the target location (but the opposite spatial relationship between the two patch stimuli locations was maintained). These patch stimuli were orthogonal to the patch stimuli in the bright/dark patch condition on 40% of trials and only these trials were included as the neutral condition for behavioral analysis because the distance between the patch location and the central fixation spot was the same as that in the bright/dark condition. Participants were informed that these patch stimuli were task-irrelevant, and thus should be ignored. Trials were included for analysis if the two variable delay times both exceeded 2000 ms (times between target and patch stimulus appearance, and between patch stimulus appearance and FP disappearance, over 75% of trials). Stimulus location and patch condition (bright, dark, and neutral) were randomly interleaved, and there were 32 trials in the bright or dark condition.

*Memory-delay saccade task (Exp 1: Figure 1B)*. The identical configuration was used as in the visual-delay saccade task (165 trials) except that the target was briefly presented for 400 ms, and participants were required to generate a saccade towards the remembered location of the target only after the disappearance of FP. Similar to the visual-delay task, trials were included for analysis if the two variable delay times both exceeded 2000 ms (over 75% of trials), and participants had to remember the target location to perform the task accurately. Note that a previous study in humans has shown similar fMRI activation between memory-delay saccade and memory-delay recognition tasks (button press response), suggesting that the sustained activation in frontal and parietal areas during the memory delay period in the memory-delay saccade was more related to working memory rather than the planning of eye movements (Srimal and Curtis, 2008).

*Visual-delay and Memory-delay saccade task with no contingency between patch location and target location (Exp 2)*. We further performed a variant for the visual-delay saccade task and memory-delay saccade task (6 participants) in which the location of the patches were equally random relative to the location of the target (while the opposite spatial relationship between the two patch stimulus locations was maintained), with 8 possible locations (4 different radial angles at 45, 135, 225, and 315°, each at two eccentricities of 4 or 8° visual angle from the central fixation spot). As such, the three patch stimulus conditions (bright, dark, and neutral) made up 12.5%, 12.5%, and 75% of the 256 trials (25% for orthogonal patch) experiment respectively. We also performed a variant of the memory-delay saccade task with 16 possible target locations (512 trials, 4 participants) to verify that the results of the eight target location variant was not due to changes in subject strategy with the reduced number of target locations. The same results were obtained, and thus the 8 and 16 possible target memory-delay tasks were combined for data analysis. There were 32 trials in the bright or dark condition. After exclusion, there were at least 10 correct trials in each condition (bright and dark) for pupillary analysis from each participant.

*Data analysis*. To maintain an accurate measure of pupil size, trials with an eye position deviation of more than 2° from the central FP or with detected saccades (>2°) during the required period of central fixation were excluded from analysis. When blinks were detected, following the literature, pre- and post-blink pupil values were used to perform a linear interpolation to replace pupil values during the blink period (Karatekin et al., 2010; Nassar et al., 2012; Wang et al., 2016). Trials were discarded when two blinks occurred within a time interval of less than 500 ms. The above criteria resulted in the removal of 8.8 and 9.0% of trials in the visual-delay and memory-delay task (Exp 1), respectively, and 2.2 and 3.1% of trials in the no contingency visual- and memory-delay task (Exp 2), respectively. Saccade reaction time (SRT) was defined as the time from the FP disappearance to the first saccade away from central fixation that exceeded 30°/s. Saccade direction deviation (angular degree) determined by saccade direction theta minus target direction theta was also reported. Failure to initiate a saccade within 1000 ms after the disappearance of FP or failure to make a saccade to the correct location (within 2.5° radius around the target) were marked as errors and were removed from pupillary analysis, consisting of 19.2 and 26.4% of trials in the visual-delay and memory-delay task (Exp 1), respectively (3.7 and 15.5% of trials removed in the no contingency visual- and memory-delay tasks- Exp 2). In Experiment 1, due to insufficient number of correct trials (<10 trials/condition) for analyses, one human participant was excluded in the visual-delay saccade task (N = 19), and three human participants were excluded in the memory-delay saccade task (N = 17). There were at least 10 correct trials in each condition (bright and dark) for included participants.

Following the procedures of baseline-correction used previously (Wang et al., 2012, 2014), for each trial a baseline pupil value was determined by averaging pupil size from 200 ms before to 50 ms after the onset of the patch presentation because the human stimulus-evoked pupil response latency is regularly longer than 150 ms (Wang and Munoz, 2014; Wang et al., 2017). Pupil values were subtracted from this baseline value. The pupil diameter value was defined as the average pupil values during the time window of 200 to 800 ms that captures the pupillary changes evoked by the patch presentation.

To specifically examine our hypothesis that pupillary responses evoked by patch stimuli should be smaller when the bright patch stimulus (compared to dark) was spatially aligned with the target location, we performed a one-sided *t* test on corresponding pupillary responses, except where indicated. Moreover, a Bayesian t test was performed to inform statistical significance for pairwise comparisons, with a scale factor r = 0.707 (Rouder et al., 2009). Note that the neutral condition was mainly used as catch trials to prevent subjects from developing a particular strategy during the tasks (e.g., expectation of the presentation of the patch stimuli at the saccade goal location or simply linking the patch locations to the target location). Therefore, the results from these trials were not of theoretical interests and not included for statistical analyses, except for behavioral data (only orthogonal patch stimulus condition) where a one way repeated-measure ANOVA was performed.

## Results

### Exp 1: Enhanced pupillary luminance responses to stimulus appearing at the saccade planning location

**Figure 2:**
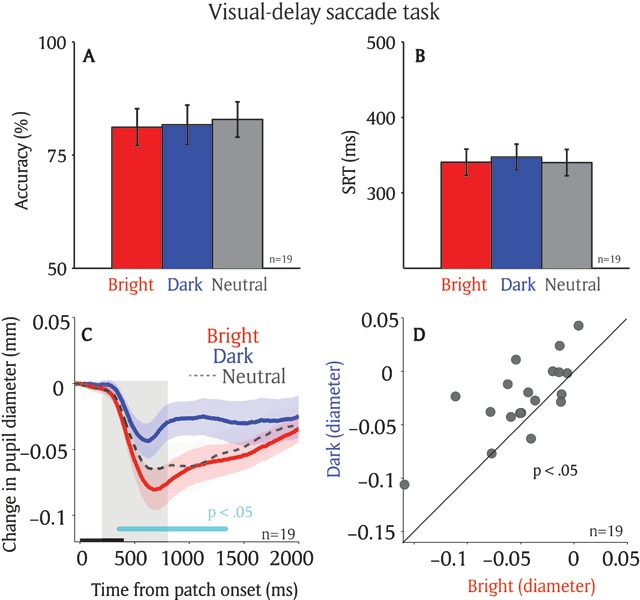
**Effect of saccade planning on the local luminance modulation.** Saccadic behaviors on different patch conditions on the visual-delay task (n = 19) **(A)** Saccade accuracy. **(B)** Saccade reaction times. **(C)** Change in pupil diameter following the presentation of patch stimuli in different conditions. **(D)** Pupil size in diameter (200 to 800 ms) between bright and dark patch conditions for each individual participant. In A, B, the error-bars represent ± standard error across participants. In C, the shaded colored regions surrounding the pupillary response represent ± standard error range (across participants) for different conditions. The black bar on X-axis indicates the time line of patch presentation, and the cyan bar on X-axis indicates the time line at which differences between the bright and dark conditions were statistically significant (*p* < 0.05). The 200–800 ms epoch after the patch onset is shaded in gray. n: number of participants. SRT: saccade reaction times.

Participants performed the visual-delay saccade task accurately with correct responses made for 81% of trials in the bright condition, 82% of trials in the dark condition, and 83% of trials in the neutral condition (Figure 2A, F(2,36) = 0.526, *p* = 0.59). Mean SRT were 341 ms in the bright condition, 348 ms in the dark condition, and 340 ms in the neutral condition (Figure 2B, F(2,36) = 0.36, *p* = 0.68). Saccade direction deviation (saccade direction relative to target direction, see Materials and Methods) were also similar among different conditions, with a mean saccade size and normalized saccade direction of 5.87 and –12.7 deg in the bright condition, 5.75 and –10.9 deg in the dark condition, and 5.72 and –12.54 deg in the neutral condition (supplementary Figure 1A, B: saccade size: F(2,36) = 0.57, *p* = 0.57; saccade direction deviation: F(2,36) = 0.11, *p* = 0.9). Moreover, the distance between eye position and target location (eye position minus target location) was similar in different conditions (supplementary Figure 1C, D: epoch of –100 to 0 ms relative to patch onset, F(2,36) = 0.81, *p* = 0.45). The mean distances between eye position and target location were 6.33, 6.08, and 6.24 deg in the bright, dark, and neutral conditions, respectively, indicating that the eyes were slightly deviated away from the subsequent target location (it should be 6 deg distance away from the target location if there is no deviation). Note that the neutral condition was mainly used as catch trials to prevent subjects from developing a particular strategy, and the theoretical focus of the current study is the pupil responses between the bright and dark conditions.

If saccade planning automatically biases visual processing at the prepared location, then pupil size evoked by the patch presentation should be smaller when the bright patch stimulus is spatially aligned with the saccade destination, as compared to the dark patch stimulus. Figure 2C illustrates changes in pupil size normalized to the patch onset pupil size on correct trials (see Materials and Methods). Pupillary constriction was evoked shortly after the presentation of patch stimuli across all conditions (Figure 2C), consistent with a previous study (Binda et al., 2013). More importantly, saccade planning modulated pupillary responses evoked by the patch stimuli, with greater constriction when the bright patch stimulus was presented at an upcoming saccadic location, compared to the dark patch stimulus. The pupil response latency (significant differences between the bright and dark conditions) was 389 ms after patch presentation (*p* < .05 denoted by a green horizontal bar in Figure 2C). As shown in Figure 2D, pupil size was significantly smaller in the bright compared to the dark condition (*t*(18) = 3.49, *p* < 0.005, BF = 16.15; epoch of post-presentation 200 ms to 800 ms). Moreover, because the eyes were deviated slightly away from the target location at the onset of patch presentation (supplementary Figure 1C, D), the observed patch effects cannot not be explained by the eye position bias. That is, the patch presented at the saccade target would be closer to the fixation point and induce a stronger pupil response.

### Exp 1: Enhanced pupillary luminance responses to stimulus appearing at a remembered location

**Figure 3:**
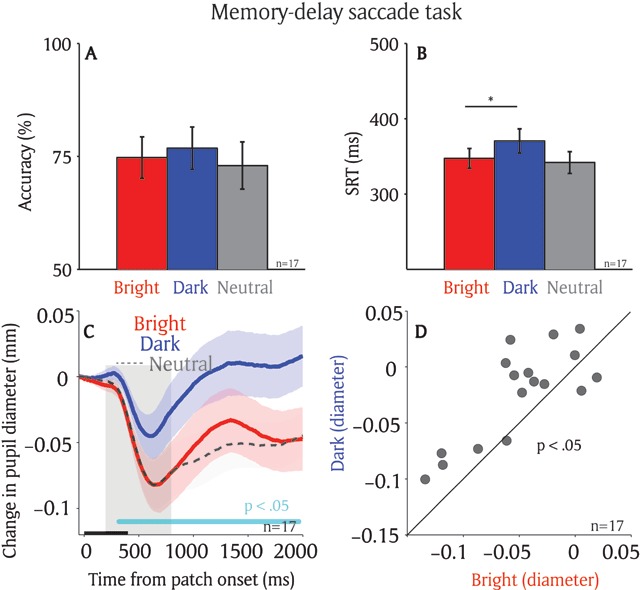
**Effect of spatial working memory on the local luminance modulation.** Saccadic behaviors on different patch conditions on the memory-delay task (n = 17) **(A)** Saccade accuracy. **(B)** Saccade reaction times. **(C)** Change in pupil diameter following the presentation of patch stimuli in different conditions. **(D)** Pupil size in diameter (200 to 800 ms) between bright and dark patch conditions for each individual participant. In A, B, the error-bars represent ± standard error across participants. * indicates the differences were statistically significant (*p* < 0.05). In C, the shaded colored regions surrounding the pupillary response represent ± standard error range (across participants) for different conditions. The black bar on X-axis indicates the time line of patch presentation, and the cyan bar on X-axis indicates the time line at which differences between the bright and dark conditions were statistically significant (*p* < 0.05). The 200–800 ms epoch after the patch onset is shaded in gray. n: number of participants. SRT: saccade reaction times.

Participants performed well in the memory-delay saccade task, with accuracies of 75% in the bright, 77% in the dark, and 73% in the neutral condition, respectively (Figure 3A, F(2,32) = 1.35, *p* = 0.27). Mean SRT were 347 ms in the bright condition, 371 ms in the dark condition, and 342 ms in the neutral condition (Figure 3B, F(2,32) = 5.92, *p* < 0.01; Bonferroni post hoc test between bright and dark condition, *p* = 0.051). Saccade size and saccade direction deviation were similar among different conditions, with a mean saccade size and saccade direction deviation of 5.77 and –13.74 deg in the bright condition, 5.72 and –11.67 deg in the dark condition, and 5.59 and –12.73 deg in the neutral condition (supplementary Figure 2A, B: saccade size: F(2,32) = 0.39, *p* = 0.68; saccade direction deviation: F(2,32) = 0.12, *p* = 0.89). Moreover, the distance between eye position and target location was similar in different conditions (supplementary Figure 2C, D, epoch of –100 to 0 ms relative to patch onset, F(2,32) = 0.43, *p* = 0.65). The mean distances between eye position and target location were 6.24, 6.12, and 6.11 deg in the bright, dark, and neutral conditions, respectively.

If spatial working memory automatically biases visual processing at the remembered location, pupil size after the patch presentation should be smaller when the bright patch stimulus, compared to the dark patch stimulus, is spatially aligned with the remembered location. Figure 3C illustrates pupillary responses recorded on correct trials in different conditions. Similar to results in the visual-delay task, pupillary constriction was evoked shortly after the presentation of patch stimuli across all conditions. Pupillary responses evoked by the patch stimuli were modulated by the remembered location. Greater pupil constriction was observed when the bright patch stimulus, compared to the dark patch stimulus, was presented at the remembered location (Figure 3C). The pupil response latency was 347 ms after patch presentation (significant differences between the bright and dark conditions denoted by a green horizontal bar in Figure 3C). As shown in Figure 3D, pupil size was significantly smaller in the bright condition compared to the dark condition (*t*(16) = 3.7, *p* < 0.005, BF = 24.12; epoch of post-presentation 200 ms to 800 ms).

### Exp 1: Comparing local luminance modulation between visual-delay and memory-delay tasks

**Figure 4:**
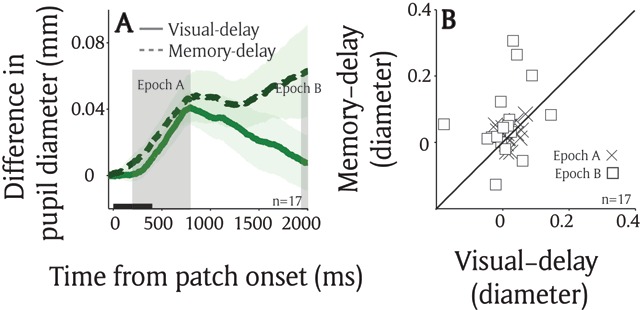
**Comparison between saccade planning and working memory effects on the local luminance modulation. (A)** Differences in pupil size between the bright and dark condition in visual-delay and memory-delay saccade tasks (n = 17). **(B)** The mean pupil size at epoch A (200 to 800 ms) and B (1950 to 2000 ms). In A, the shaded colored regions surrounding the pupillary response represent ± standard error range (across participants) for different tasks. The black bar on X-axis indicates the time line of patch presentation. n: number of participants.

Although the local luminance modulation by pre-saccadic processes were pronounced in both the visual-delay and memory-delay saccade tasks, there were differences between two tasks. Figure 4A illustrates differences in pupil size between the bright and dark conditions in the two tasks in the same participants (N = 17), showing similar local luminance modulations in the transient epoch (Figure 4B: post-presentation 200 ms to 800 ms, *t*(16) = 0.917, *p* > 0.3, BF = 0.36). Although not significant, during the sustained epoch, there was a slightly larger local luminance modulation in the memory-delay task, compared to the visual-delay task (Figure 4B: post-presentation 1950 ms to 2000 ms, *t*(16) = 2.04, *p* = 0.05, BF = 1.31), implying local luminance effects mediated by saccade planning and working memory may not be completely identical.

**Figure 5:**
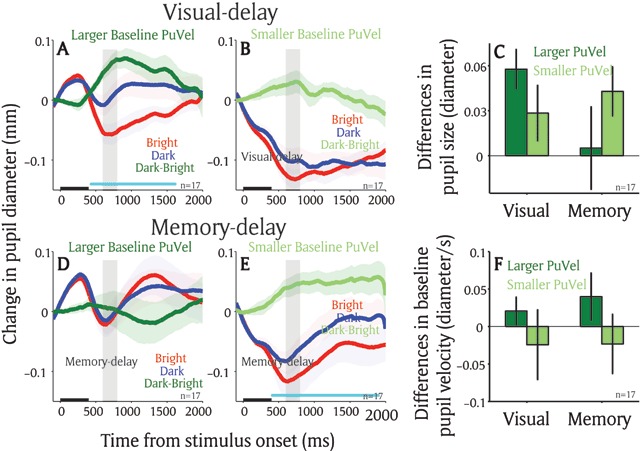
**Arousal effect on the local luminance modulation between two tasks.** Change in pupil diameter following the presentation of patch stimuli in different conditions in the visual-delay saccade task (n = 17) in larger baseline (–50 to 0 ms) pupil velocity **(A)**, or smaller baseline pupil velocity **(B)**. Change in pupil diameter following the presentation of patch stimuli in different conditions in the memory-delay saccade task (n = 17) in larger baseline (–50 to 0 ms) pupil velocity **(D)**, or smaller baseline pupil velocity **(E)**. **(C)** Pupil size in diameter (600 to 800 ms) between bright and dark patch conditions as a function of task (visual-delay and memory-delay) and baseline pupil velocity (larger and smaller). **(F)** Baseline pupil velocity (–50 to 0 ms) between bright and dark patch conditions as a function of task (visual-delay and memory-delay) and baseline pupil velocity (larger and smaller). In A, B, D, E, the shaded colored regions surrounding the pupillary response represent ± standard error range (across participants) for different conditions. The black bar on X-axis indicates the time line of patch presentation, and the cyan bar on X-axis indicates the time line at which differences between the bright and dark conditions were statistically significant (*p* < 0.05). n: number of participants. Difference: differences between the bright and dark conditions. PuVel: pupil velocity. Visual: visual-delay task. Memory: memory-delay task.

To further investigate the potential differences between the two tasks, we examined the influence of brain state (or arousal) on the local luminance modulation. Trials were separated according to higher and lower pupil velocity (median split) at the time of patch presentation (baseline epoch: averaged from 50 ms before to the patch stimulus onset), as the rate of change in pupil size is often used to index arousal level (Aston-Jones and Cohen, 2005; Nassar et al., 2012; Eldar et al., 2013; de Gee et al., 2014; Reimer et al., 2014; Ebitz and Platt, 2015). In the visual-delay task, while in the stage of higher arousal (higher baseline pupil velocities), pupil size after the patch presentation was significantly smaller when the target location was spatially aligned with the bright patch as compared to the dark patch stimulus (Figure 5A: post-presentation 600 ms to 800 ms, *t*(16) = 4.55, *p* < 0.01, BF = 99.74). However, these effects were reduced in the stage of lower arousal (Figure 5B: post-presentation 600 ms to 800 ms, *t*(16) = 1.6, *p* = 0.065, BF = 0.72). In contrast, in the memory-delay task, with higher arousal (higher baseline pupil velocities), pupil size after the patch presentation was similar between the bright and dark conditions (Figure 5D: post-presentation 600 ms to 800 ms, *t*(16) = 0.19, *p* > 0.4, BF = 0.25), whereas the local luminance modulation was pronounced in lower arousal stage (Figure 5E: post-presentation 600 ms to 800 ms, *t*(16) = 2.71, *p* < 0.01, BF = 3.76). The interaction between the local luminance modulation (differences in pupil size between bright and dark conditions) and tasks (visual-delay and memory-delay) was statistically significant (2 × 2 repeated measure ANOVA: F(1,16) = 2.74, *p* < 0.05 in Figure 5C, all other *ps* > 0.3). These effects cannot be attributed to the differences in the baseline pupil velocity because the interaction between baseline pupil velocity (differences in rate of pupil size change between bright and dark conditions) and tasks (visual-delay and memory-delay) was not statistically significant (2 × 2 repeated measure ANOVA: F(1,16) = 0.303, *p* > 0.5 in Figure 5F, all other *ps* > 0.2). Future research is required to explore these differences to understand similarities and differences among different preparatory processes on this modulation.

### Exp 2: Local luminance modulation when there was no contingency between patch and target locations

Although pupil responses evoked by the patch presentation were biased toward the target location in both tasks, the patch stimuli were related to a subsequent target location because a target was presented at one of the patch locations on 40% of the trials. To explore whether the modulation is still pronounced when the patch stimuli are completely uninformative to the subsequent target location, the same tasks with no contingency between patch and target locations were conducted (i.e. patch stimuli could appear at any possible target locations in a given target location condition with equal probability, see Materials and Methods). Participants performed well in both tasks with accuracies and mean SRTs of 94% and 315 ms in the bright condition, 98% and 310 ms in the dark condition, and 96% and 307 ms in the neutral condition in the visual-delay task (N = 6; see supplementary Figure 3 for other eye movement data). In the memory-delay task, accuracies and mean SRTs were 86% and 298 ms in the bright condition, 85% and 301 ms in the dark condition, and 83% and 307 ms in the neutral condition (N = 10; see supplementary Figure 4 for other eye movement data).

**Figure 6:**
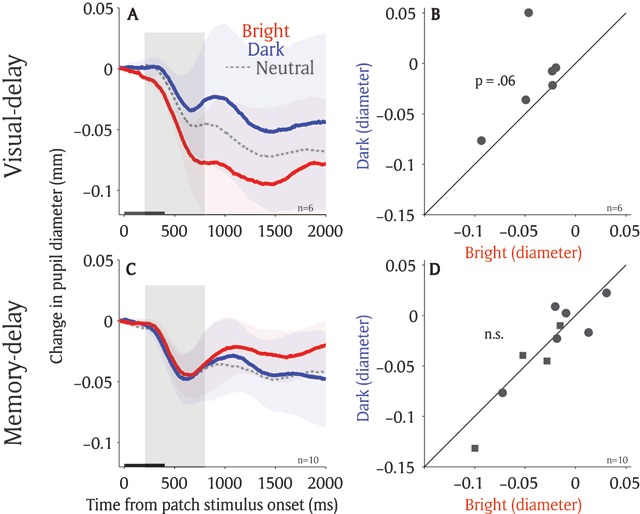
**Local luminance modulation with no contingency between patch and target location. (A)** Change in pupil diameter following the presentation of patch stimuli in different conditions in visual-delay saccade task. **(B)** Pupil size in diameter (200 to 800 ms) between bright and dark patch conditions for each individual participant on the visual-delay saccade task (n = 6). **(C)** Change in pupil diameter following the presentation of patch stimuli in different conditions in memory-delay saccade task. **(D)** Pupil size in diameter (200 to 800 ms) between bright and dark patch conditions for each individual participant on the memory-delay saccade task (n = 10). In A, C, the shaded colored regions surrounding the pupillary response represent ± standard error range (across participants) for different conditions. The black bar on X-axis indicates the time line of patch presentation. In D, circles indicate the data collected in the experiment with 8 possible target locations whereas squares indicate the data collected in the experiment with 16 possible target locations. n: number of participants.

Similar to previous experiments, pupillary responses evoked by the patch stimuli were modulated by the target location in the visual-delay task. Greater pupil constriction was observed when the bright patch stimulus, compared to the dark patch stimulus, was presented at an upcoming saccadic location (Figure 6A). As shown in Figure 6B, pupil size was marginally smaller in the bright condition compared to dark condition (*t*(5) = 1.85, *p* = 0.06, BF = 1.1; epoch of post-presentation 200 ms to 800 ms). The effects were diminished, however, in the memory-delay task (Figure 6C). Pupil size in the bright condition was similar to that in the dark condition (*t*(9) = 0.6, *p* = 0.72, BF = 0.36; epoch of post-presentation 200 ms to 800 ms).

## Discussion

Spatial attention is an essential cognitive function that enables observers to select and bias processing of information toward a selected location, resulting in faster and better processing at that location compared to others (Carrasco, 2011; Maunsell, 2015; Moore and Zirnsak, 2017). Similar effects have been observed at the location being prepared for upcoming saccades or maintained in working memory, suggesting that spatial attention is guided to the locations programmed for upcoming saccades or maintained in working memory (Awh and Jonides, 2001; Awh et al., 2006; Bisley, 2011; Kowler, 2011). Here, we compared the effects of saccade planning and working memory by simultaneously presenting bright and dark luminance patches during the delay period in the visual-delay and memory-delay tasks. We found significantly greater pupil constriction after presentation of the bright patch, compared to the dark patch, at the spatial location of the target in both tasks. However, there were small differences in the sustained pupil responses and arousal modulation between the two tasks. Also, the patch effects were diminished when there was no contingency implemented between the patch and target locations in the memory-delay task. Together, our results, using involuntary changes of pupil size, provide evidence suggesting similar, but not identical, modulation of saccade planning and working memory on pupil local luminance responses.

### Local-luminance modulation between saccade planning and spatial working memory

Saccade planning and working memory are interdependent with attention (Awh and Jonides, 2001; Awh et al., 2006; Bisley, 2011; Kowler, 2011; Melcher and Piazza, 2011; Hanning et al., 2016; Ohl and Rolfs, 2017), and similar neural networks are recruited during these cognitive operations (Ikkai and Curtis, 2011; Jerde et al., 2012). Given this interdependent relationship, it has been suggested that the location prepared for eye movements or maintained in working memory attracts spatial attention (Rizzolatti et al., 1987; Soto et al., 2008). Our results support this idea. Because of the deployment of spatial attention for saccade planning or working memory, pupil size following patch presentation was smaller when the bright patch, compared to the dark patch, was spatially aligned with the target location (Figures 2 and 3). The observed local luminance modulation was similar to that obtained through allocation of spatial attention (Binda et al., 2013, 2014; Mathôt et al., 2013; Mathôt et al., 2014; Binda and Murray, 2015b), saccade planning, or working memory (Mathôt et al., 2015; Fabius et al., 2017; Unsworth and Robison, 2017, 2018), together suggesting the mediation of attentional mechanisms on this behavior.

Although the local luminance effect was observed in the visual-delay and memory-delay tasks, there were differences in the local luminance modulation between the two tasks. First, the sustained responses between the bright and dark condition in the memory-delay task were marginally larger than those in the visual-delay task (Figure 4). Moreover, arousal modulation, as indicated by instantaneous pupil velocity, was different between two tasks (Figure 5). More importantly, these local luminance effects were diminished in the memory-delay task when the contingency between the patch and a subsequent target locations was eliminated (Figure 6), suggesting that the modulation is pronounced only when the patch locations are associated with the target location (the target appeared at one of the patch locations on 40% of trials in the first experiment). This observation is remotely similar to the notion that not all working memory items guide attention (Olivers et al., 2011). That is, a working memory item that is relevant to the task guides the deployment of attention, whereas an item that is not relevant to the task at hand does not. These results together suggest that although saccade planning and working memory both involve attention, their involvements in attention are not identical. Future research is required to explore the effects of arousal and task context on these two cognitive operations, as well as their relationship to spatial attention.

### Neural mechanisms underlying the local luminance modulation

What neural substrates mediate this behavior? The midbrain superior colliculus (SC), a phylogenetically old structure, is a subcortical sensorimotor center for saccadic eye movements (Hall and Moschovakis, 2003; Gandhi and Katnani, 2011; White and Munoz, 2011; Krauzlis et al., 2013) that receives important cognitive signals from the gaze and attention network regions such as the frontal eye field and lateral intraparietal cortex (Wardak et al., 2004, 2006; Thompson and Bichot, 2005; Bisley and Goldberg, 2010; Krauzlis et al., 2013). The intermediate layers of the SC (SCi) integrate inputs from visual areas, frontal-parietal regions, and basal ganglia, and projects directly to the premotor circuit in the brainstem to initiate the orienting response, including not only shifts of attention and gaze, but also pupil dilation (Corneil and Munoz, 2014; Wang and Munoz, 2015).

Because the SCi receives convergent cognitive signals from cortical and subcortical areas, it is importantly involved in various cognitive operations such as spatial attention, saccade preparation, and working memory. Inactivation of the SC diminishes the ability of monkeys to select useful visual information presented at the location corresponding to an affected SC site for perceptual judgement (Lovejoy and Krauzlis, 2010; Zenon and Krauzlis, 2012), or for target selection in a visual search task (McPeek and Keller, 2004). Saccade preparation increases SCi activity in the neurons corresponding to the saccade destination, and the level of increased activity correlates with the likelihood of the saccade being made to that location (Dorris et al., 1997, 2007; Dorris and Munoz, 1998). Moreover, it has been shown that the SCi activity is sustained during the delay period in both visual-delay and memory-delay saccade tasks (Glimcher and Sparks, 1992; Kojima et al., 1996; Pare and Wurtz, 2001), arguably through the signals from the frontal eye field, lateral intraparietal cortex, dorsal lateral prefrontal cortex, supplementary eye field, and basal ganglia (Pare and Wurtz, 1997; Hikosaka et al., 2000; Sommer and Wurtz, 2000; Wurtz et al., 2001; Johnston and Everling, 2009; Stuphorn et al., 2010). In summary, the SCi is involved in various *pre-saccadic* processes, including spatial attention, saccade preparation, and spatial working memory, and is also critical in integrating and relaying signals from the frontal eye field and lateral intraparietal cortex to the pupil control circuit to mediate the local luminance modulation.

## Conclusion

Spatial attention is regularly linked to saccade planning and spatial working memory. Here, we demonstrated a similar modulation of local pupil luminance responses at the location being prepared for an upcoming saccade or maintained in working memory, which was also similar to the response mediated by spatial attention. These results suggest that the mechanisms involved in spatial attention are also initiated in the visual-delay and memory-delay tasks, and further highlight the promising potential of using this paradigm for objectively probing visuospatial processing.

## Additional Files

The additional files for this article can be found as follows:

10.5334/joc.33.s1 Supplementary Figure 1. Saccadic behaviors on the visual-delay task (Exp 1) in different patch conditions.

10.5334/joc.33.s1 Supplementary Figure 2. Saccadic behaviors on the memory-delay task (Exp 1) in different patch conditions.

10.5334/joc.33.s1 Supplementary Figure 3. Saccadic behaviors on the no-contingency visual-delay task (Exp 2) in different patch conditions.

10.5334/joc.33.s1 Supplementary Figure 4. Saccadic behaviors on the no-contingency memory-delay task (Exp 2) in different patch conditions.

10.5334/joc.33.s2 Data. General interface dynamics, button functionality, and hyperlink descriptions.

## Data Availability

All raw data is included in the Additional Files section.
