# Supplementary material for: Comparing Pupil Light Response Modulation between Saccade Planning and Working Memory
Source: J Cogn. 2018 Jun 26;1(1):33. doi: 10.5334/joc.33 (PMC6634341; doi:10.5334/joc.33)
Supplement: Supplementary Figure 1. — Saccadic behaviors on the visual-delay task (Exp 1) in different patch conditions. [file joc-1-1-33-s1.pdf]

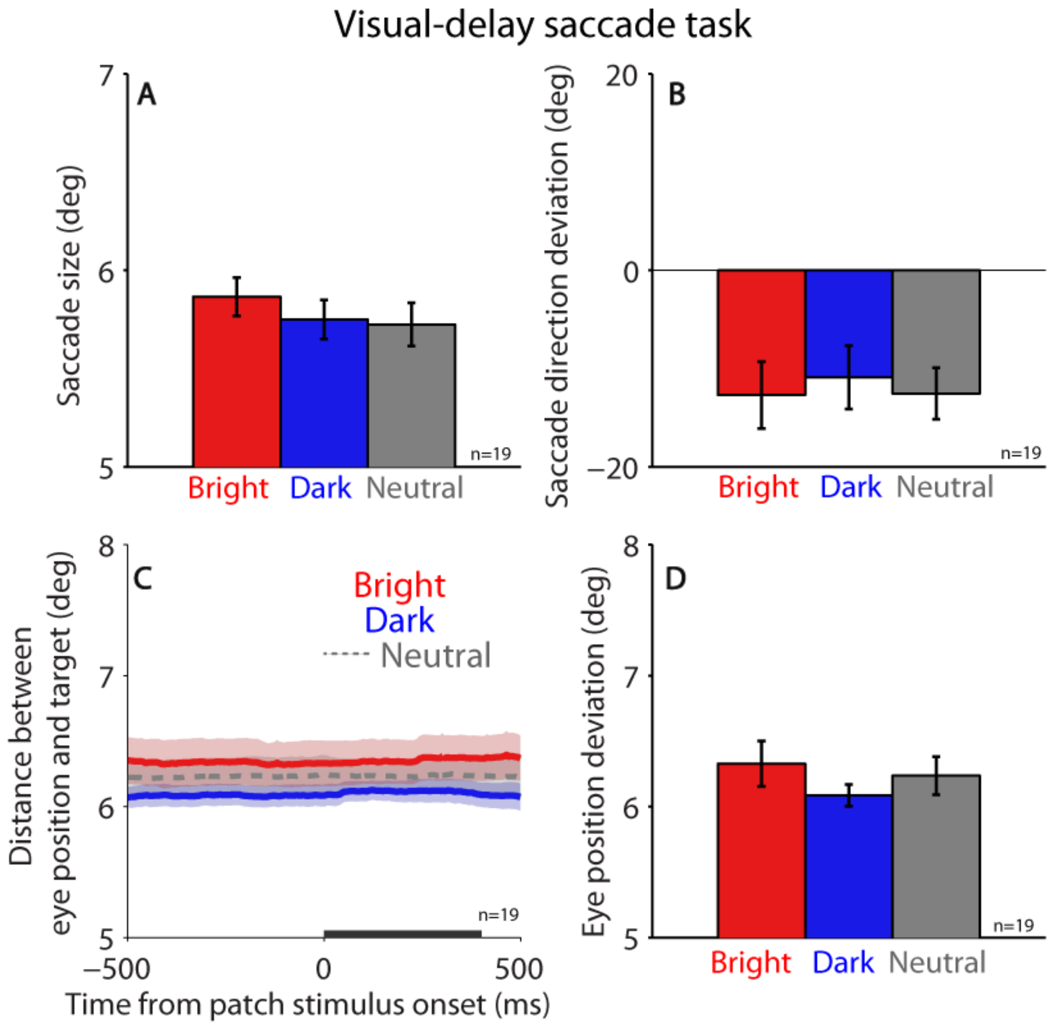

Supplementary Figure 1. Saccadic behaviors on the visual-delay task (Exp 1) in different patch conditions. (A) Saccade size. (B) Saccade direction deviation (angular degree: saccade direction minus target direction). (C) Distance between eye position and target location following the presentation of patch stimuli in different conditions. (D) Eye position deviation at patch onset (-100 to 0 ms) in different conditions. In A,B,D, the error-bars represent ± standard error across participants. In C, the shaded colored regions surrounding eye position traces represent ± standard error range (across participants) for different conditions. The black bar on X-axis indicates the time line of patch presentation. n: number of participants.

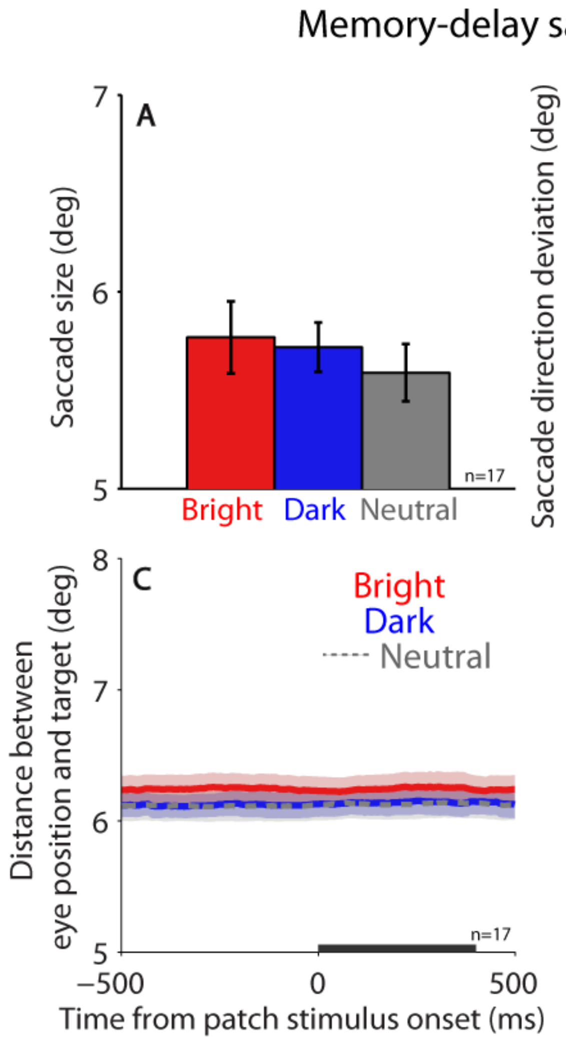

Supplementary Figure 2. Saccadic behaviors on the memory-delay task (Exp 1) in different patch conditions. (A) Saccade size. (B) Saccade direction deviation. (C) Distance between eye position and target location following the presentation of patch stimuli in different conditions. (D) Eye position deviation at patch onset (-100 to 0 ms) in different conditions. In A,B,D, the error-bars represent ± standard error across participants. In C, the shaded colored regions surrounding eye position traces represent ± standard error range (across participants) for different conditions. The black bar on X-axis indicates the time line of patch presentation. n: number of participants..

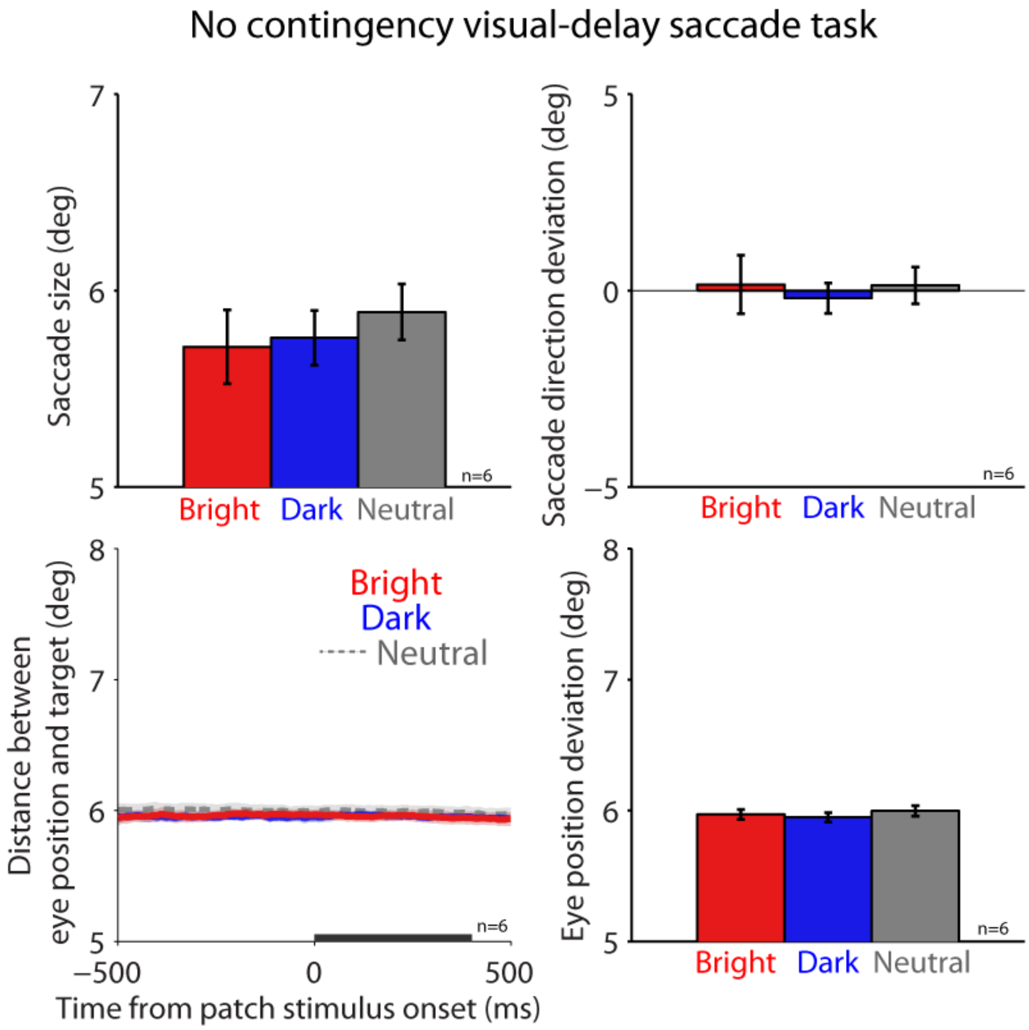

Supplementary Figure 3. Saccadic behaviors on the no-contingency visual-delay task (Exp 2) in different patch conditions. (A) Saccade size (5.71, 5.76, 5.89 deg in the bright, dark, and neutral condition, respectively, $F(2,10) = 0.41$, $p = 0.67$). (B) Saccade direction deviation (0.16, -0.19, 0.13 deg in the bright, dark, and neutral condition, respectively, $F(2,10) = 0.15$, $p = 0.86$). (C) Distance between eye position and target location following the presentation of patch stimuli in different conditions. (D) Eye position deviation at patch onset (-100 to 0 ms) in different conditions (5.97, 5.95, and 6 deg in the bright, dark, and neutral condition, respectively, $F(2,10) = 0.47$, $p = 0.63$). In A,B,D, the error-bars represent ± standard error across participants. In C, the shaded colored regions surrounding eye position traces represent ± standard error range (across participants) for different conditions. The black bar on X-axis indicates the time line of patch presentation. n: number of participants.

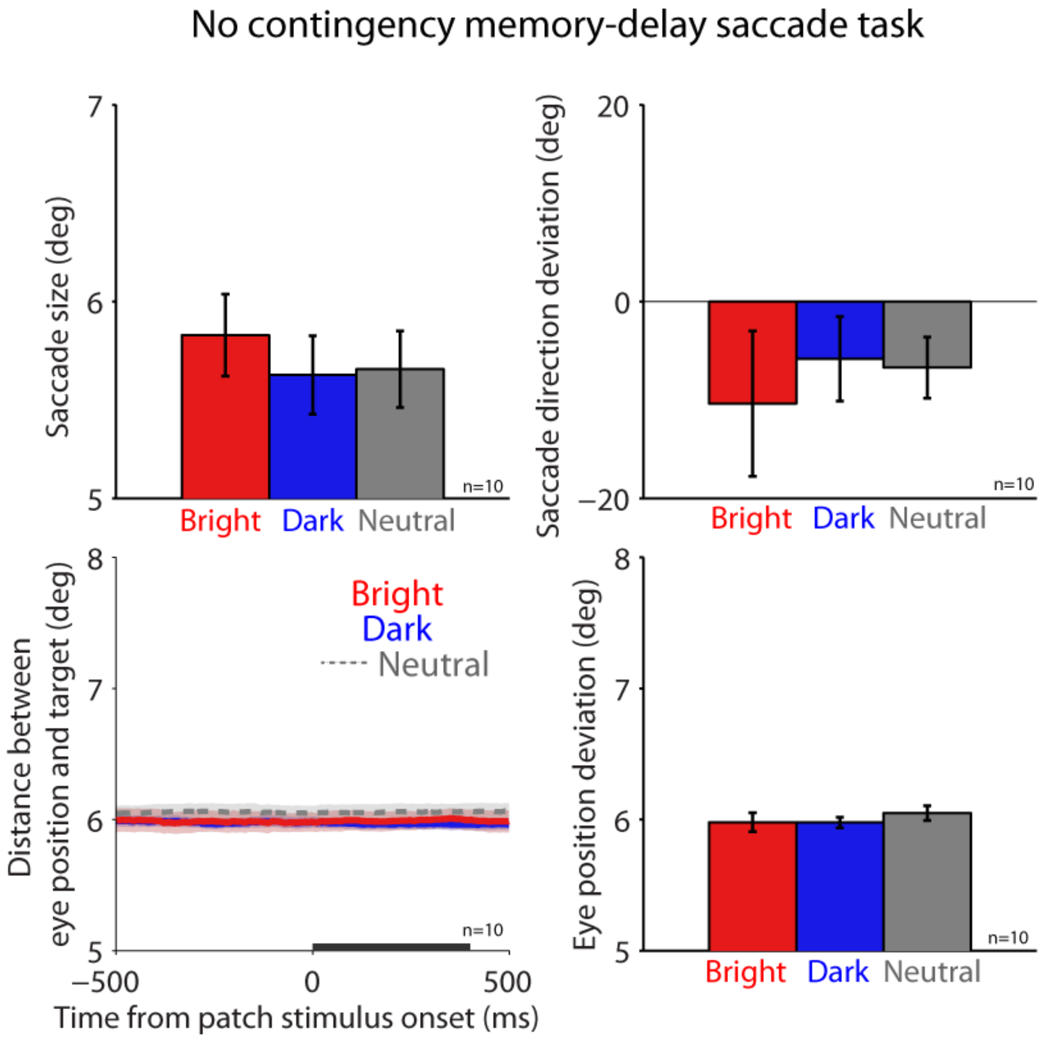

Supplementary Figure 4. Saccadic behaviors on the no-contingency memory-delay task (Exp 2) in different patch conditions. (A) Saccade size (5.83, 5.63, 5.66 deg in the bright, dark, and neutral condition, respectively, F(2, 18) = 0.33, p = 0.72). (B) Saccade direction deviation -10.39, -5.82, -6.71 deg in the bright, dark, and neutral condition, respectively, : F(2, 18) = 0.24, p = 0.79). (C) Distance between eye position and target location following the presentation of patch stimuli in different conditions. (D) Eye position deviation at patch onset (-100 to 0 ms) in different conditions (5.98, 5.98, and 6.05 deg in the bright, dark, and neutral condition, respectively, F(2,18) = 0.54, p = 0.59). In A,B,D, the error-bars represent ± standard error across participants. In C, the shaded colored regions surrounding eye position traces represent ± standard error range (across participants) for different conditions. The black bar on X-axis indicates the time line of patch presentation. n: number of participants.
